# Natural bioactive gases: immunomodulatory properties and effects on human physiology of plant-derived biogenic volatile organic compounds and phytoncides

**DOI:** 10.4103/mgr.MEDGASRES-D-26-00076

**Published:** 2026-06-17

**Authors:** Mohammad Mahdi Asaadi, Giovanni N. Roviello

**Affiliations:** Department of Molecular Medicine and Medical Biotechnology, University of Naples Federico II, Naples, Italy; Institute of Biostructures and Bioimaging, National Research Council (IBB-CNR), Naples, Italy

##  

**Forest-derived volatile compounds as drivers of human physiological modulation:** Immersive experiences in green settings, particularly forests, have long been associated with beneficial effects on human health, including reduction in stress, favorable changes in mood, and improvements in cardiovascular and immune systems.^1^ These outcomes have traditionally been attributed to a complex interplay of acoustic, visual, behavioral, and social factors. However, an increasing body of evidence suggests that inhalation of plant-emitted biogenic volatile organic compounds (BVOCs) and phytoncides may represent a specific, quantifiable biological mechanism contributing to these positive effects.

BVOCs such as α-pinene, limonene, and other monoterpenes are continuously released into the atmosphere by different species of trees and other plants, shaping the chemical composition of forest air (**Figure 1**), especially during the warm months. Recent observational and interventional studies have increasingly linked the inhalation of these compounds to measurable improvements in anxiety, immune function, as well as physiological stress markers. For example, in a large observational cohort of more than 500 participants undergoing structured forest therapy sessions at multiple Italian sites, higher exposure to inhaled monoterpenes was associated with a statistically significant reduction in state anxiety scores, with α-pinene emerging as a key contributor to the anxiolytic effect.^1^ These findings support the concept that specific volatile molecules present in forest air can exert targeted psychophysiological mechanisms.

**Figure 1:**
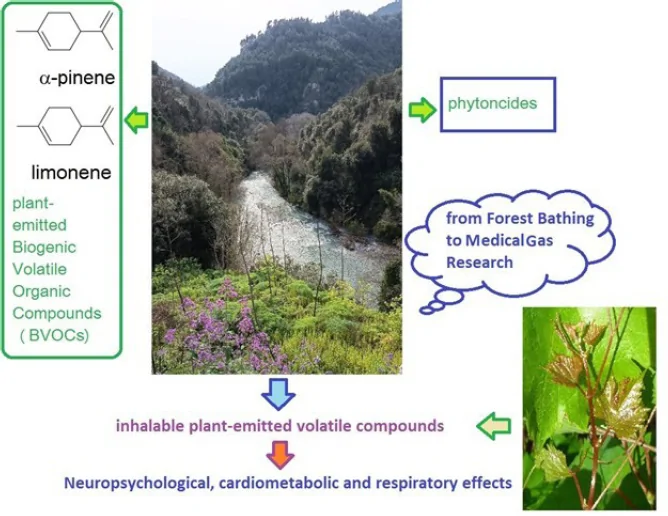
Potential applications of plant-emitted BVOCs and phytoncides. Photo provided by G.N. Roviello. BVOC: Biogenic volatile organic compound.

Clinical and preclinical studies on inhaled essential oils, of which monoterpenes are major constituents, have concurrently demonstrated significant effects on anxiety, depression, and stress-related outcomes in humans and animal models. Proposed mechanisms include systemic absorption via the cardiorespiratory system with subsequent delivery to the central nervous system, as well as direct modulation of limbic circuits through olfactory pathways.^1^,^2^,^3^ These dual mechanisms mirror, at least conceptually, the systemic and local effects observed with several established medical gases.

Medical gas has traditionally referred to small inorganic or simple gaseous molecules such as oxygen, nitric oxide, carbon dioxide, hydrogen, hydrogen sulfide, xenon, and others, administered in controlled concentrations for diagnostic or therapeutic purposes. These gases are recognized for their ability to modulate vascular tone, inflammation, oxidative stress, neurotransmission, and cellular signaling. However, the broader scope of the discipline increasingly encompasses gasotransmitters, gaseous signaling molecules, and gas–biomaterial interactions, emphasizing function, bioactive gaseous modulation of physiology, over strict chemical class.^4^

The plant-derived BVOCs and phytoncides can be conceptually framed as natural bioactive gases. The volatile molecules present in the inhaled atmosphere, at sufficient concentrations and exposure durations, can modulate immune responses, neuroendocrine function, and psychological states. Although they differ chemically from classical medical gases, being larger in structure and more chemically complex organic compounds, their mode of delivery (inhalation), systemic distribution, and capacity to influence certain physiological processes align them with the functional definition of medical gas–like agents.

Herein, we examine the evidence supporting immunomodulatory and broader physiological effects of plant-derived BVOCs and phytoncides in humans and relevant models, and discuss whether, and under what conditions, these compounds could be considered medical gases.

**Plant-derived BVOCs in function-oriented therapy:** Linking traditional medical gases with plant volatiles involves a structural paradox. While gases (such as oxygen, carbon dioxide, hydrogen, nitrous oxide, xenon), and the gasotransmitters (such as nitric oxide and hydrogen sulfide) are simple, plant-derived BVOCs have complex structures.^5^ As studies demonstrate for medical gases, these molecules effectively cross biological barriers to modulate neuro-immune signaling.^6^,^7^,^8^ Similarly, current literature highlights that plant-derived volatiles share this capacity, directly impacting the central nervous system.^2^,^3^ By defining these volatiles as natural bioactive gases, we shift the focus from molecular size to physiological function, integrating them into the medical gas framework.

BVOCs consist of various secondary metabolites, such as monoterpenes (C_10_H_16_), sesquiterpenes (C_15_H_24_), and their oxygenated derivatives. While the term “phytoncides” was originally coined for antimicrobial properties, its modern use refers to tree-emitted volatiles that support human health through inter-organismal signaling. Key compounds like α-pinene, β-pinene, and limonene possess high volatility and lipophilicity. According to Antonelli et al.^3^ these physicochemical traits facilitate efficient alveolar absorption and the crossing of the blood–brain barrier following inhalation. In forest environments, concentrations of these volatiles range from 1 to 10 ppbv (parts per billion by volume), significantly higher than in urban settings where anthropogenic pollutants dominate. Understanding these dynamic emission patterns is essential for replicating forest-like atmospheres in controlled clinical environments to achieve predictable therapeutic outcomes.

Forest therapy has been shown to improve innate immunity, mainly by activating natural killer (NK) cells and modulating cytokine levels, with spending time in forest environments increasing the count and activity of NK cells, as well as cytotoxic proteins like perforin and granzyme A. These immune benefits are quite persistent, with studies showing that NK activity can remain high for up to 30 days after a forest trip.^9^

Phytoncides, which are bioactive plant volatiles, are the primary mediators of the above-mentioned effects.^2^ Research using wood essential oils, rich in monoterpenes like α-pinene, has successfully replicated the immune responses seen in natural forests. Direct inhalation of phytoncides, as an example, induces NK cell activity and increases anti-cancer protein expression.^9^ Furthermore, Ochiai et al.^10^ found that forest walking improves mucosal immunity, specifically salivary IgA levels, more than urban walking.

The immunomodulatory role of BVOCs is similar to established medical gases like molecular H_2_, which also has anti-inflammatory and antioxidant properties.^11^ While gases like hydrogen or nitric oxide are simple signaling molecules, BVOCs act as complex “natural bioactive gases” interacting with the body’s neuro-immune network. These effects derive from neuroimmune interactions and redox modulation, and inhaling these volatiles causes a shift toward parasympathetic dominance, which lowers stress-related catecholamines that usually suppress the immune system in a process eventually building enhanced physiological resilience.^2^

**Neuropsychological, cardiometabolic and respiratory effects of inhaled BVOCs:** Observational studies confirm that inhaling forest monoterpenes like α-pinene reduces state anxiety, measured by the State-Trait Anxiety Inventory (STAI-S). Donelli et al.^1^ noted that this follows a dose-response relationship; higher atmospheric BVOC levels lead to better psychological restoration. Systematic reviews also show that forest immersion lowers cortisol, the main stress biomarker. Mechanistically, these volatiles activate olfactory–limbic pathways, promoting parasympathetic dominance. By stabilizing the neuroendocrine system and improving heart rate variability, these compounds turn forest settings into ‘healing environments’ where environmental factors directly enhance mental health resilience.^2^

Forest bathing leads to important cardioprotective benefits by modulating the hemodynamic and respiratory systems. Research shows that spending time in forests, and thus, inhaling BVOCs, is associated with a lower blood pressure, a slower heart rate, and a better heart rate variability. These natural bioactive gases also boost respiratory health.^2^ Specifically, Li et al.^12^ proved that in people at risk of chronic obstructive pulmonary disease, forest exposure improves oxygen saturation and decreases inflammatory markers.

Recent literature highlights that these findings align with the effects of traditional medical gases like nitric oxide and hydrogen.^2^ While nitric oxide works as a direct vasodilator, inhaled compounds like α-pinene regulate blood vessels by shifting the autonomic balance toward the parasympathetic system and reducing stress hormones. Passively breathing these volatiles thus represents a useful strategy for reducing cardiovascular risk and managing stress-related metabolic issues.^2^ Integrating these environmental factors into preventive medicine offers an affordable, scalable way to handle the health challenges of urban life and aging.

Remarkably, the link between BVOCs and medical gases rests on their therapeutic convergence. Since the anti-inflammatory and neurobehavioral effects of these volatiles overlap with those of certain traditional medical gases, such as hydrogen and xenon,^6^,^7^ we suggest defining medical gases by how they modulate physiological functions via inhalation. This medical gas–like classification helps standardize organic volatiles within research, bridging the gap between natural environments and clinical medicine.^2^

**Standardized BVOCs and next-generation inhalation platforms:** Standardizing plant-derived volatiles for inhalation therapy fits well with current advancements in medical gas research. We propose using enriched BVOCs as adjunctive treatments for anxiety, chronic inflammation, and supportive oncology care.

Future research must address gaps in dose-response data, safety, and randomized trials.^2^ We propose multidisciplinary chamber studies to validate BVOCs against conventional medical gases. Integrating in silico studies with real-time monitoring is key to identifying mechanistic pathways. However, central questions remain regarding the ability of artificial atmospheres to reach the therapeutic thresholds observed in natural forests, and the extent to which direct chemical effects can be distinguished from broader environmental and psychological influences. We also expect the development of BVOC-based nasal sprays and medical devices capable of releasing controlled pressures and concentrations of these volatile therapeutic molecules in the coming years.

Regarding safety, while these compounds are therapeutic at forest-level concentrations, higher doses must be carefully evaluated; as concentrated volatile compounds, particularly terpenes such as limonene and α-pinene, can exhibit dose-dependent cytotoxicity or trigger irritant responses.^13^,^14^

**Conclusion:** Plant-derived BVOCs and phytoncides form a pervasive yet underexploited class of inhalable airborne bioactive molecules with demonstrable effects on human physiology, particularly in the domains of immunomodulation, stress and anxiety reduction, and potentially cardiometabolic regulation. Although they differ fundamentally from classical medical gases in chemical structure and origin, their mode of delivery via inhalation, their capacity to modulate cellular and tissue functions, and their emerging translational potential justify considering them within an expanded, function-oriented framework of medical gas research. The available evidence, including large observational cohorts and clinical studies on essential oils, supports the view that both naturally occurring exposures in green environments and artificially enriched or pressurized plant-emitted volatile compound mixtures could be harnessed for therapeutic purposes, if composition, dosing, and safety are rigorously controlled. While this conceptual framework is promising, it remains largely hypothesis-generating and requires further experimental validation. At the same time, deep chemical and mechanistic differences from conventional medical gases impose clear conceptual and regulatory boundaries, and any analogy must be drawn with caution. We propose that plant-derived BVOCs and phytoncides be regarded as a distinct subclass of natural bioactive gases, medical gas–like agents, whose systematic investigation may open new avenues for non-invasive, environment-inspired interventions in neuro-immune and stress-related therapies.

*The authors declare that no Generative AI was used in the preparation of this manuscript.*
